# Eight-and-a-Half Syndrome: A Case Report on a Rare Pontine Neuro-Ophthalmologic Presentation

**DOI:** 10.7759/cureus.103604

**Published:** 2026-02-14

**Authors:** Lalit Kumar Tyagi, Mansi Singh

**Affiliations:** 1 General Medicine, Santosh Medical College and Hospital, Santosh Deemed To Be University, Ghaziabad, IND; 2 General Medicine, American International Institute of Medical Sciences, Udaipur, IND

**Keywords:** eight-and-a-half syndrome, facial nerve palsy, internuclear ophthalmoplegia, paramedian pontine reticular formation, pontine infarction

## Abstract

Eight-and-a-half syndrome is a distinctive neuro-ophthalmologic presentation resulting from a focal pontine lesion that simultaneously disrupts horizontal gaze pathways, internuclear connections, and facial nerve fibers. Clinically, it manifests as horizontal gaze palsy, internuclear ophthalmoplegia, and ipsilateral lower motor neuron facial weakness. We report a case of an elderly man with vascular risk factors who presented with the inability to perform conjugate horizontal gaze, adduction deficit of one eye with abducting nystagmus of the contralateral eye, and ipsilateral facial palsy, while vertical eye movements were preserved. Magnetic resonance imaging of the brain revealed an acute pontine infarction correlating with the neurological findings. This case highlights the importance of recognizing this characteristic constellation of signs for accurate anatomical localization and diagnosis.

## Introduction

Eight-and-a-half syndrome is an uncommon brainstem condition defined by the coexistence of a horizontal gaze disorder, internuclear ophthalmoplegia, and ipsilateral peripheral facial nerve palsy [1,2]. The syndrome reflects the involvement of multiple adjacent neural structures within the pontine tegmentum responsible for ocular motility and facial movement [3]. Previously reported cases in the literature have described this syndrome in association with diverse etiologies, most commonly pontine infarction, but also demyelinating diseases, infectious and inflammatory processes, and neoplastic lesions [4-11]. Comparative analyses of these cases suggest that ischemic lesions tend to present abruptly and demonstrate good clinicoradiological correlation, whereas non-vascular causes may show more variable clinical courses. Recognition of this characteristic clinical pattern is important, as it allows precise anatomical localization and facilitates early identification of the underlying pathology.

## Case presentation

A 71-year-old, right-handed male with a background of hypertension and ischemic heart disease, on regular treatment, presented with an abrupt onset of dizziness, slurred speech, diplopia on rightward gaze, and deviation of the mouth toward the right. He denied tobacco or alcohol use and reported no limb weakness, sensory symptoms, imbalance, headache, or prior cerebrovascular events.

**Figure 1:**
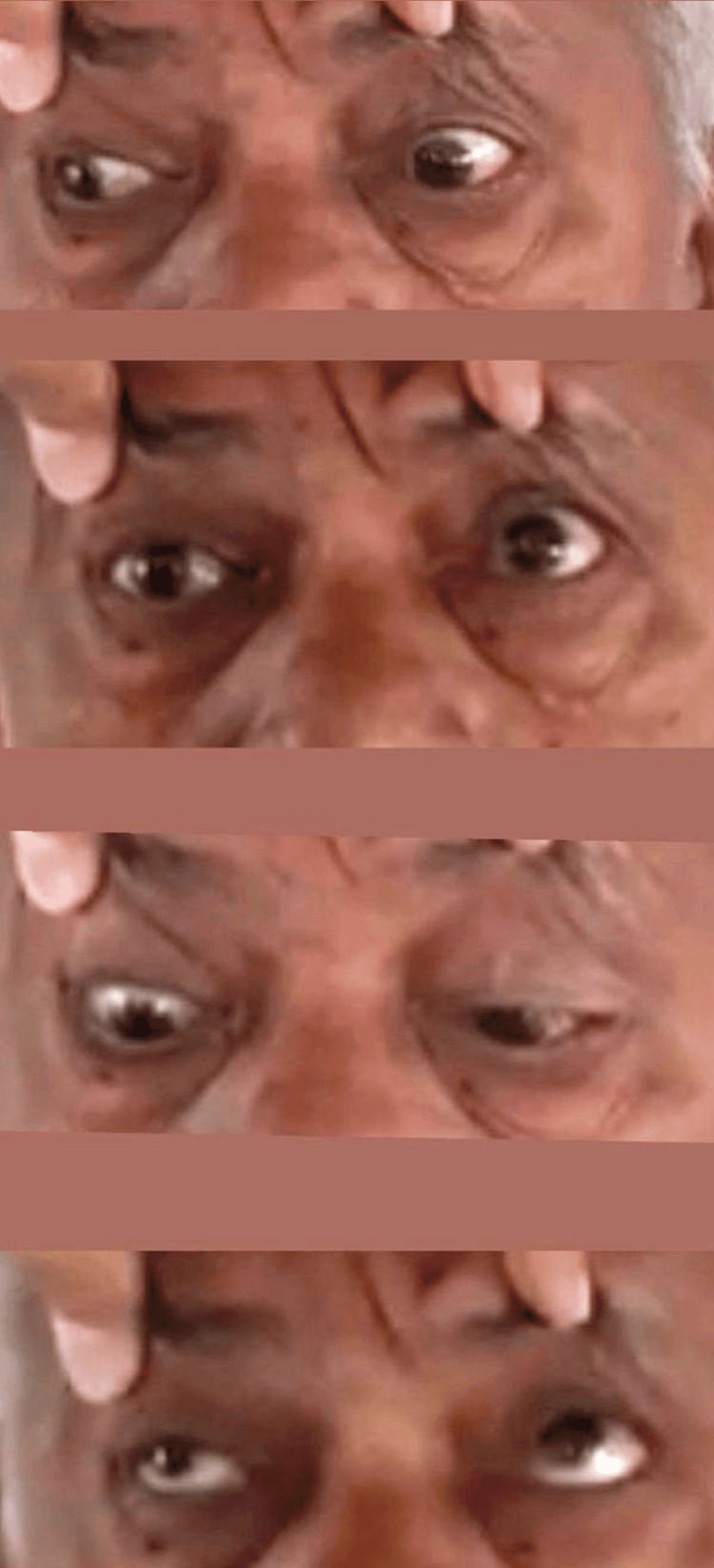
(a) On attempted right gaze, there is failure of adduction of the left eye with preserved abduction of the right eye, consistent with ipsilateral internuclear ophthalmoplegia due to medial longitudinal fasciculus involvement. (b) Primary gaze demonstrates near-central ocular alignment; precise assessment is limited by the absence of a clearly visible corneal light reflex. (c) and (d) Vertical gaze (upward and downward) is preserved. Ipsilateral lower motor neuron facial palsy was noted on clinical examination, characterized by a loss of forehead wrinkling, incomplete eye closure, flattening of the nasolabial fold, and deviation of the angle of the mouth (image not shown). Patient-identifiable features have been removed to maintain confidentiality.

Neurological examination revealed outward deviation of the right eye in the primary position. On attempted gaze to the left, there was a complete failure of horizontal eye movement with absent saccades. Rightward gaze demonstrated preserved abduction of the right eye with accompanying gaze-evoked nystagmus (Figure 1). Vertical eye movements and vertical saccades were intact, and the vestibulo-ocular reflex was preserved. A left-sided lower motor neuron facial nerve palsy with Bell’s phenomenon was noted. No ptosis, pupillary abnormality, or involvement of other cranial nerves was observed. Motor, sensory, cerebellar, and meningeal examinations were normal.

**Figure 2:**
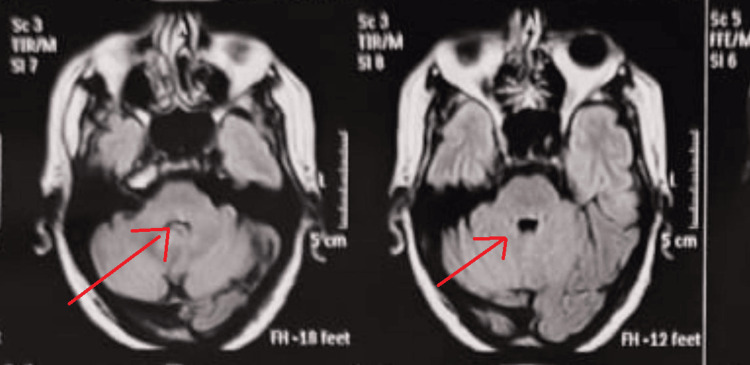
MRI demonstrates a small diffusion-restricted infarct in the left dorsal pons, hyperintense on DWI/FLAIR with corresponding ADC hypointensity and no hemorrhage. While individual brainstem nuclei are not distinctly visualized, the lesion location supports the clinical diagnosis of eight-and-a-half syndrome. DWI: diffusion-weighted imaging; FLAIR: fluid-attenuated inversion recovery; ADC: antibody–drug conjugate

Magnetic resonance imaging of the brain showed an acute diffusion-restricted lesion involving the left dorsal pons. The anatomical location of the infarct corresponded to structures governing horizontal gaze and facial nerve function, thereby supporting the diagnosis of eight-and-a-half syndrome (Figure 2). Vertebral artery Doppler study revealed mild intimal thickening without significant stenosis, and transthoracic echocardiography demonstrated preserved left ventricular systolic function.

## Discussion

Eight-and-a-half syndrome arises from a single pontine lesion that simultaneously affects multiple functionally related neural pathways. Disruption of the paramedian pontine reticular formation or abducens nucleus produces horizontal gaze palsy, involvement of the medial longitudinal fasciculus results in internuclear ophthalmoplegia, and damage to the facial nerve fascicle at the level of the facial colliculus leads to ipsilateral lower motor neuron facial weakness [1,3,12]. Together, these deficits create a characteristic and highly localizable clinical pattern.

Ischemic injury to the pons, particularly involving perforating branches of the vertebrobasilar circulation, represents a common mechanism underlying this syndrome [3-5,12-16]. Previously reported cases have demonstrated that vascular etiologies typically present with an abrupt onset of symptoms and show close clinicoradiological correlation, whereas demyelinating, infectious, inflammatory, or neoplastic causes often follow a more subacute or progressive course and may be associated with additional neurological findings.

Neuroimaging plays a crucial role in supporting the diagnosis and identifying the underlying etiology. Diffusion-weighted magnetic resonance imaging is particularly valuable in detecting acute pontine infarction, as small strategically located lesions can produce disproportionate clinical deficits due to the dense concentration of neural pathways within the pons. In non-vascular etiologies, contrast-enhanced imaging may further aid in differentiation.

Clinical recovery may be variable and often begins with improvement in facial weakness, followed by gradual partial recovery of ocular motility, although residual deficits can persist in cases of vascular origin [13-16]. The extent of recovery largely depends on lesion size, underlying pathology, and early initiation of appropriate management, including vascular risk factor modification and neurorehabilitation.

This case highlights the importance of careful bedside neuro-ophthalmologic examination in patients presenting with ocular motility disturbances and facial weakness. Early recognition of eight-and-a-half syndrome allows prompt anatomical localization, narrows the differential diagnosis, and facilitates targeted investigations and timely treatment, thereby improving clinical outcomes.

## Conclusions

Although eight-and-a-half syndrome is rare, it should be considered in elderly patients with vascular risk factors who present with acute diplopia and facial asymmetry. Careful ocular motility assessment combined with targeted neuroimaging is essential for accurate diagnosis.
